# Electrostatic-field and surface-shape similarity for virtual screening and pose prediction

**DOI:** 10.1007/s10822-019-00236-6

**Published:** 2019-10-24

**Authors:** Ann E. Cleves, Stephen R. Johnson, Ajay N. Jain

**Affiliations:** 1 Applied Science, BioPharmics LLC, Santa Rosa, CA USA; 2 Computer-Assisted Drug-Design, Bristol-Myers Squibb, Co., Princeton, NJ USA; 3 Dept. of Bioengineering and Therapeutic Sciences, University of California, San Francisco, USA

**Keywords:** Molecular similarity, Virtual-screening, Pose prediction, Surflex, ForceGen, ROCS, Ligand-based modeling, Ligand alignment

## Abstract

We introduce a new method for rapid computation of 3D molecular similarity that combines electrostatic field comparison with comparison of molecular surface-shape and directional hydrogen-bonding preferences (called “eSim”). Rather than employing heuristic “colors” or user-defined molecular feature types to represent conformation-dependent molecular electrostatics, eSim calculates the similarity of the electrostatic fields of two molecules (in addition to shape and hydrogen-bonding). We present detailed virtual screening performance data on the standard 102 target DUD-E set. In its moderately fast screening mode, eSim running on a single computing core is capable of processing over 60 molecules per second. In this mode, eSim performed significantly better than all alternate methods for which full DUD-E data were available (mean ROC area of 0.74, p $< 10^{-9}$, by paired t-test, compared with the best performing alternate method). In addition, for 92 targets of the DUD-E set where multiple ligand-bound crystal structures were available, screening performance was assessed using alternate ligands or sets thereof (in their bound poses) as similarity targets. Using the joint alignment of five ligands for each protein target, mean ROC area exceeded 0.82 for the 92 targets. Design-focused application of ligand similarity methods depends on accurate predictions of geometric molecular relationships. We comprehensively assessed pose prediction accuracy by curating nearly 400,000 bound ligand pose pairs across the DUD-E targets. Overall, beginning from agnostic initial poses, we observed an 80% success rate for RMSD $\le 2.0$ Å  among the top 20 predicted eSim poses. These examples were split roughly 50/50 into cases with high direct atomic overlap (where a shared scaffold exists between a pair) and low direct atomic overlap (where where a ligand pair has dissimilar scaffolds but largely occupies the same space). Within the high direct atomic overlap subset, the pose prediction success rate was 93%. For the more challenging subset (where dissimilar scaffolds are to be aligned), the success rate was 70%. The eSim approach enables both large-scale screening and rational design of ligands and is rooted in physically meaningful, non-heuristic, molecular comparisons.

## Introduction

Calculation of 3D ligand similarity has become a widely used approach within computer-aided drug design, especially for virtual screening but also for pose prediction and multiple ligand alignment. Many different methods have been developed, based on aspects of volumetric overlap, surface concordance, or matching of electrostatic or other ligand features such as aromatic rings. The field has been the subject of extensive review, and best practices for the benchmarking of such methods has been the subject of a special issue published in this journal. We refer interested readers to the perspective from Nicholls et al. for a review [1] and the collection of papers published here in 2008 [2], which address numerous aspects of virtual screening assessment, pose prediction, statistical evaluation approaches, and benchmark construction [3–13].

**Fig. 1:**
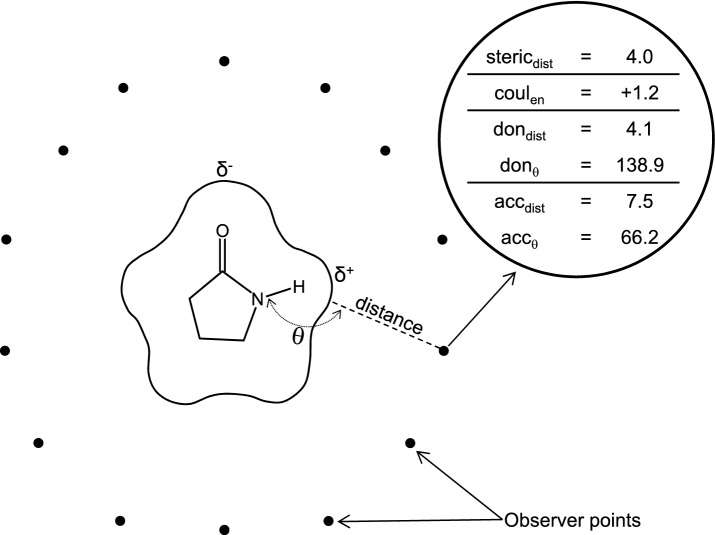
A 2D depiction of the eSim feature calculation shows 2-pyrrolidone surrounded by 14 observer points at which each of six values are computed, all of which depend on the exact conformation and alignment of the subject molecule

Here, we introduce a new 3D similarity method that combines the surface-based approach of Surflex-Sim and related methods [14–16] with an electrostatic field comparison method that derives from the recently introduced QuanSA 3D-QSAR approach [17]. The method is called “eSim” for electrostatic-field and surface-shape similarity (with the three “s” characters giving rise to the capital “S”). Figure 1 depicts the central calculation within eSim, which is the computation of feature values at observer points, with the latter having been placed outside of a query molecule (also referred to as a “target molecule” in what follows). At each observer point, six values are computed:

1. $\textit{steric}_{dist}$: The distance in Angstroms from the point to the atom surface, which corresponds to the minimum over the distances to each atom less that atom’s VdW radius.
2. $\textit{coul}_{en}$: The molecular electric field is characterized in terms of the Coulombic energy of moving a point charge of + 0.2*e* to the observer point from an infinite distance (as in the QuanSA approach [17], with additional details provided in the Methods Section).
3. $\textit{don}_{dist}$: The minimum distance from the observer to any donor proton’s surface.
4. $\textit{don}_{theta}$: The angle formed by the observer point, the donor proton, and the atom attached to the donor proton.
5. $\textit{acc}_{dist}$: The minimum distance from the observer to any acceptor atom’s surface.
6. $\textit{acc}_{theta}$: The angle formed by the observer point, the acceptor atom, and the centroid of the atoms attached to the acceptor atom (180°, for example, when a pyridine nitrogen is oriented perfectly toward an observer).

**Fig. 2:**
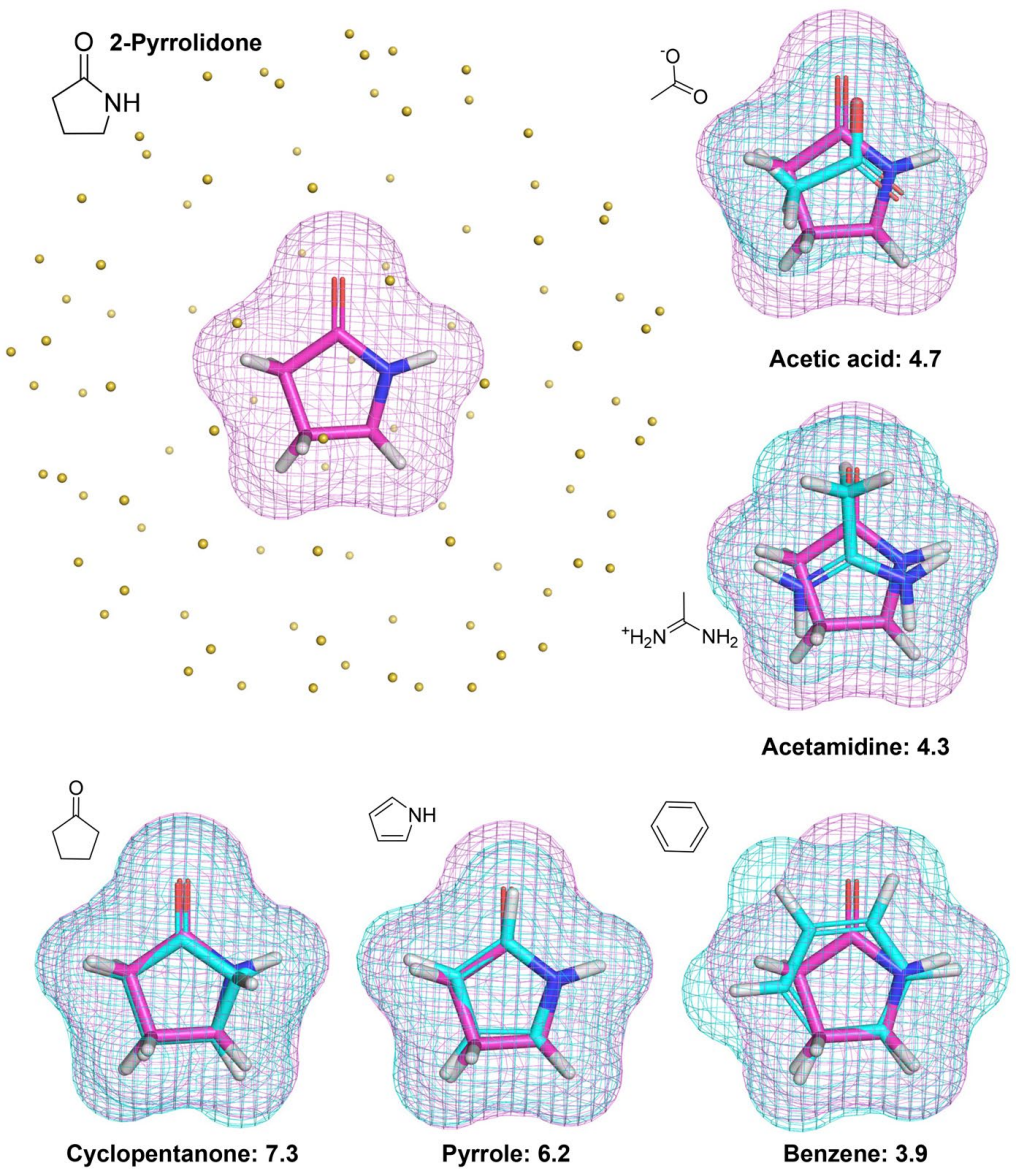
Five small molecules (cyan) are shown in their optimal poses relative to the query/target 2-pyrrolidone (magenta, shown with yellow observer points, upper left), with their corresponding eSim scores

Given the pose of a subject molecule, aligned within the query molecule’s observer points, the similarity of the two is computed using a sum of Gaussian functions of the differences between the respective feature values. If, for each observer point, the subject molecule is able to mimic the query molecule perfectly, the differences will all be 0, the corresponding Gaussian function values will all be 1, and the normalized eSim score of the comparison will be 10.0 (minus the estimate of ligand strain on the subject molecule). The precise details of the similarity function will be given in the Methods Section, but the key features are that it is: (1) responsive to differences in molecular surface shape without being directly dependent on atomic center correspondence in measuring shape; (2) responsive to both the distance and direction of hydrogen bond participants; (3) capable of detecting differences in the local electrostatic fields of molecules that may otherwise be close to isosteric and contain identical hydrogen bonding features; and (4) continuous and piecewise differentiable with respect to molecular pose.

Figure 2 shows the 2-pyrrolidone example in 3D (magenta) along with the optimal alignments and eSim scores of five small molecules (cyan), each with differences in the disposition of surface shape, charge, and hydrogen bonding. In terms of the relative ranking, cyclopentanone is the most closely isosteric, and it mimics the acceptor functionality of the target. Pyrrole also mimics a single hydrogen-bonding atom of the target, but it is less perfectly isosteric and, owing to its aromaticity, is also less ideal a match in terms of the electrostatic field comparison. Both acetic acid and acetamidine have formal charges, which the target lacks, but they mimic the target better in some respects than benzene, which does not match the hydrogen bonding or electrostatics of the target and only partially mimics the surface shape.

The DUD-E docking benchmark [18] has become a *de facto* standard for measuring virtual screening effectiveness for both docking methods and ligand similarity methods. The benchmark covers a wide variety of pharmaceutically relevant targets (102 total), each with a curated protein structure and cognate bound ligand, along with a set of active ligands and computationally generated decoys. Two recent reports [19, 20] make use of the full set, as given, taking the DUD-E crystallographic ligand as the “query” in virtual screens, to benchmark molecular similarity methods in terms of both accuracy and speed. The similarity methods included cover widely-used methods such as ROCS [21, 22], other volumetric approaches (VAMS [19], WEGA [23], and OptiPharm [20]), and approaches that utilize conformer-specific features to characterize shape (USR [24]).

**Fig. 3:**
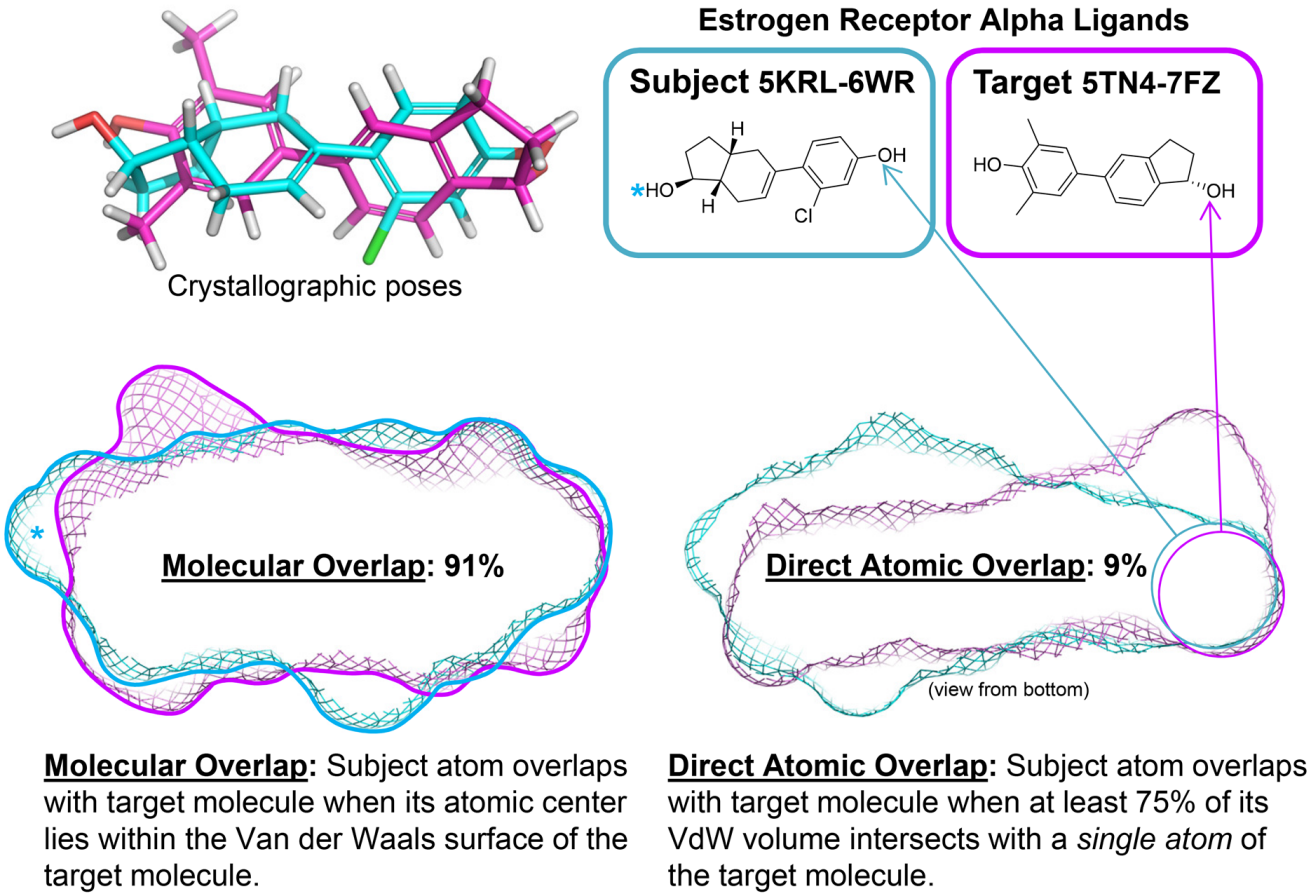
Two estrogen receptor ligands in the bound poses (subject molecule shown in cyan and target shown in magenta, upper left), exhibit nearly total molecular overlap in terms of the coverage of the 5KRL ligand by the 5TN4 ligand, but there is almost no direct atomic overlap between the two

The performance of the eSim method for virtual screening will be demonstrated using the full DUD-E benchmark to facilitate comparisons of virtual screening efficiency and calculation speed with OptiPharm, ROCS, ROCS-shape, USR, USR-shape, VAMS, and WEGA. A number of reports involving new methods have either made use of various subsets of the DUD-E targets, actives, or decoys, employed machine-learning to produce target-specific tuned performance, or have made use of crystallographic information to augment ligand similarity computations; these methods will not be discussed here, owing to the difficulty in making meaningful comparisons.

We have also curated an augmentation to DUD-E that consists of, on average, over 50 aligned protein structures and corresponding bound ligands per target. This is the largest set of structure-based multiple alignments for testing ligand similarity of which we are aware. It will be used to characterize the effect of varying query molecules on similarity-based screening as well as for extensively characterizing pose-prediction. We call this augmented data set the “DUD-E$^+$” benchmark.

In characterizing pose prediction for ligand-based approaches, we consider pairs of molecules in their aligned crystallographic poses. One is designated as the target, and the other the subject, for which a memory-free configuration is generated and then is prepared using thorough conformational search (see [25, 26] for details). Just as with docking, there is an easy form of this task and a hard form. The easy form (analogous to cognate ligand re-docking) is characterized by a high degree of substructural similarity between the subject and target molecules, where a high degree of direct atomic overlap between the two is observed in the crystallographic poses. The hard form (analogous to cross-docking) occurs when relatively little such atom-centric correspondence exists. However, for ligand similarity to be expected to work at all, the subject molecule must share some significant level of physical overlap with the target.

We define two measures of overlap, direct atomic overlap (DAO) and molecular overlap (MO), which distinguish tractable cases from intractable ones and distinguish between the easier and harder of the alignment challenges. Figure 3 illustrates the distinction between the two. For each measure, the percentage of atoms on the subject molecule that meet the measure’s criterion is calculated. For MO, a subject atom passes if its atomic center falls anywhere within the Van der Waals volume of the target molecule. In Fig. 3, the two estrogen receptor ligands are shown in the bound poses, and the MO is 91%, with just three atoms of the subject molecule protruding from within the target (including the hydroxyl proton marked with an asterisk). For DAO, at least three-quarters of a subject atom’s Van der Waals volume must intersect with a *single* atom of the target molecule. In the case shown, just 9% of the subject molecule’s atoms meet this criterion (three atoms, including the hydroxyl oxygen that is noted in the Figure). Note that the definition of DAO is somewhat sensitive to minor alignment changes, but the degree of ligand alignment uncertainty derived from protein-structure pocket alignments does not interfere with reliable identification of cases with high or low DAO.

**Fig. 4:**
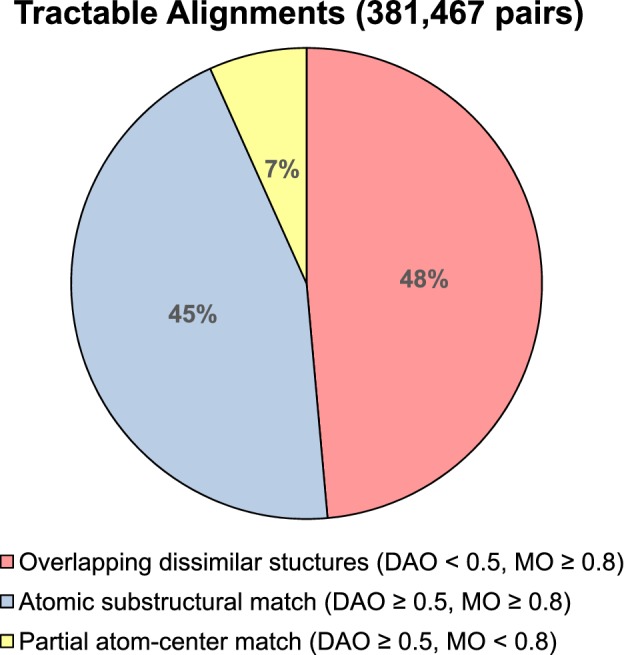
Nearly 400,000 alignment pairs exist where there is some degree of reasonable overlap from one ligand onto the other, split fairly evenly between cases with low direct atomic overlap and high

In order to thoroughly analyze the effects of query ligand choice and to comprehensively assess pose prediction, we identified all PDB structures for each DUD-E target that had a matching UniProt identifier. After an automated procedure for bond-order assignment, protonation, and mutual alignment (see Methods for details), we obtained between 5 and 359 variants for each of 92 DUD-E targets (an average of 56 bound ligands per target). We have defined the set of interesting “tractable” alignment pairs as those where: (1) the subject and target molecule are non-identical; and (2) the subject has at least 80% molecular overlap with the target *or* it has at least 50% direct atomic overlap with the target. Figure 4 shows the breakdown of the 381,467 alignment pairs that fall within this set. Overall, 93% of the cases have high molecular overlap and 52% have high direct atomic overlap. The high-MO but low-DAO subset (as in the example from Fig. 3) is nearly 50% of the total.

In what follows, we will describe the eSim method in detail as well as the curation process for the DUD-E$^+$ benchmark. Comparative results for screening enrichment will be presented for eSim, the seven alternate methods listed above, and for the related Surflex-Sim method. Using DUD-E$^+$, the effects of making use of alternate query ligands will be analyzed as well as the effect of using a *joint* query comprised of five crystallographically aligned ligands for each protein target.

The eSim method is intuitive and based on physical aspects of protein-ligand interactions, and it avoids purely heuristic molecular descriptors. Its performance, in terms of virtual screening, represents a significant advance over alternate methods, both in terms of accuracy and speed, certainly to the extent that computational benchmarks such as DUD-E can measure. Using a single computing core, eSim is capable of screening nearly 25,000,000 molecules per day, and the flexibility of the multi-core implementation allows for rapid computations involving deeper search when predicting molecular poses. The eSim method is implemented within the Similarity module of the Surflex Platform.

## Methods

Where possible, data were collected to support fair and direct comparisons between the methods reported here and widely used alternatives. Every effort has been made to ensure that the curated data fairly represents the benchmark data underpinning other published reports, and care has been taken to make use of unbiased, fully automatic, computational procedures in order to eliminate human bias. In cases where pose prediction accuracy was assessed, care was taken to remove *all* memory of 3D coordinates prior to generating initial 3D structural models and proceeding with conformational elaboration and similarity-based pose prediction. Note that all non-eSim virtual screening performance data were taken from the cited literature, where experts applied the respective methods to data sets specifically prepared for utilization by those methods.

### Similarity definition

The eSim approach is related to the morphological similarity method that was introduced in 2000 [16]. The change in eSim is the inclusion of Coulombic field comparison, but there are also some differences in the way that hydrogen bond directionality is represented, in the details of the treatment of observer points, and with the inclusion of explicit ligand strain. The overall similarity score, as a function of the subject ligand pose $L_P^s$, is given as follows:

1 $$\begin{aligned} S(L_p^s)&= \frac{\sum \nolimits _{i=1}^{n} W_i (S^{stc}_{i} + S^{coul}_{i} + S^{don}_{i} + S^{acc}_{i})}{C^{t}} + e(L_p^s) \end{aligned}$$

The sum is done over *n* observer points (see Fig. 1), with $C^{t}$ being a normalizing constant such that the comparison of the target ligand with itself yields a value of 10.0. The value $e(L_p^s)$ is a negative value based on an estimate of ligand strain (detailed below). The component functions in Eq. 1 will be defined in terms of the feature values at the observer points.

The component functions and some aspects of feature value definitions depend on Gaussian and sigmoidal functions, as follows:

2 $$\begin{aligned} g(x,\sigma )&= e^{-x^2/\sigma } \end{aligned}$$

3 $$\begin{aligned} s(x,\alpha )&= \frac{1}{1+e^{-x/\alpha }} \end{aligned}$$

When an observer point set is constructed, it is done with respect to an ideal distance from the target ligand, denoted $\gamma$ (the default value is 4.0 Å, and the procedure for placing observer points is detailed below). As outlined above, at each observer point, six values are calculated, as follows:

1. $\textit{steric}_{dist}$: The distance in Angstroms from the point to the atom surface, which corresponds to the minimum over the distances to each atom less that atom’s VdW radius. This will be denoted $stc_i^s$ for the subject ligand at observer point *i* and $stc_i^t$ for the target ligand.
2. $\textit{coul}_{en}$: The molecular electric field is characterized in terms of potential Coulombic energy, denoted $coul_i^s$ and $coul_i^t$. At each observer point, the potential energy of a point charge of 0.2e (moved from an infinite distance to the observer’s location) is calculated, with $\mathrm{r}_{ij}$ denoting the distance from observer *i* to atom *j* and the partial charge of atom *j* being $q_j$, as follows:
  4 $$\begin{aligned} d_{ij}&= r_{ij} - (\gamma - 2.0) \end{aligned}$$
  5 $$\begin{aligned} \epsilon (x)&= \frac{1}{1 - s(x - (\gamma + 2.0)),0.5)} \end{aligned}$$
  6 $$\begin{aligned} coul_i&= \sum \nolimits _{j=1}^{n} \frac{0.2 \cdot 332.0716 \cdot q_j}{\epsilon (r_{ij})(d_{ij}+0.05)} \end{aligned}$$
  Equation 4 re-scales the distances from the atoms being observed, such that the observer points feel the effects of partial atomic charges as if the observers were closer to the atomic surface, which is important in order to differentiate between spatially close differences in charge. Note that if $d_{ij}$ is less than zero, the value is clipped to zero. Equation 5 produces a value of 1 at close distances, and it begins to increase at a distance of approximately one water shell beyond $\gamma$, and then it increases without bound, becoming effectively infinite at large distances. The units of $coul_i^s$ and $coul_i^t$ are in kcal/mol (Eq. 6). By defining the Coulombic feature values in this manner, they measure local electrostatic effects primarily, and they have a computationally convenient cutoff that allows skipping of ligand atoms above a threshold distance.
3. $\textit{don}_{dist}$: The minimum distance from the observer *i* to any donor proton’s surface is denoted $don_i^s$ and $don_i^t$.
4. $\textit{don}_{theta}$: The angle formed by the observer point, the minimum-distance donor proton, and the atom attached to that donor proton is denoted $don\theta _i^s$ and $don\theta _i^t$.
5. $\textit{acc}_{dist}$: The minimum distance from the observer to any acceptor atom’s surface is denoted $acc_i^s$ and $acc_i^t$.
6. $\textit{acc}_{theta}$: The angle formed by the observer point, the minimum-distance acceptor atom, and the centroid of the atoms attached to that acceptor atom is $acc\theta _i^s$ and $acc\theta _i^t$.

For a subject ligand in a given pose to be compared to some target ligand, at each observer point *i*, Gaussian functions of the difference in observation values are computed, as follows:

7 $$\begin{aligned} S^{stc}_{i}&= g((stc_i^s - stc_i^t),\sigma _{stc}) \end{aligned}$$

8 $$\begin{aligned} S^{coul}_{i}&= g((coul_i^s - coul_i^t),\sigma _{coul}) \end{aligned}$$

9 $$\begin{aligned} S^{don}_{i}&= g((don_i^s - don_i^t),\sigma _{da}) \cdot \nonumber \\&\quad g((don\theta _i^s - don\theta _i^t),\sigma _{da\theta }) \cdot w^{don}_{i} \end{aligned}$$

10 $$\begin{aligned} S^{acc}_{i}&= g((acc_i^s - acc_i^t),\sigma _{da}) \cdot \nonumber \\&\quad g((acc\theta _i^s - acc\theta _i^t),\sigma _{da\theta }) \cdot w^{acc}_{i} \end{aligned}$$

In the case of the donor and acceptor comparisons, both the distance and directionality of the respective feature values on the subject and target ligands must match in order to yield a local similarity score of unity. Each of the $\sigma$ values is a constant that determines how sharply the Gaussians fall off with differences in the respective feature value types.

The $w_i$ values for the donor and acceptor comparisons are defined as follows:

11 $$\begin{aligned} w^{don}_{i}&= s((\gamma +2.0) - don_i^s, \alpha _{da}) + \nonumber \\&\quad s((\gamma +2.0) - don_i^t, \alpha _{da}) \end{aligned}$$

12 $$\begin{aligned} w^{acc}_{i}&= s((\gamma +2.0) - acc_i^s, \alpha _{da}) + \nonumber \\& \quad s((\gamma +2.0) - acc_i^t, \alpha _{da}) \end{aligned}$$

The $\alpha _{da}$ constant controls the steepness of the sigmoidal falloff in the $w_i$ values. This concentrates the attention of the observer points on similarity values associated with donor and acceptor atoms that are *close* to each observer. If, for example, a ligand has just a single donor, comparisons at only those observer points close to the donor will be responsive in terms of influence on similarity. These weights scale the comparisons in a similar fashion to the treatment of the dielectric, with the effects of donors and acceptors falling off through a sigmoidal step function as they become distant from the observer point.

Thus far, all aspects of the similarity calculation have been symmetric, with both the subject and target molecules being treated identically. One aspect of the calculation is not symmetric, and this has to do with both the placement of and weighting of observer points. The observer points are placed in such a fashion to be ideal with respect to the *target ligand pose* (the procedure is described in detail below). With a single target ligand, all observer points are places such that their minimum distance to the target ligand’s surface is $\gamma$. Making meaningful comparisons where one wants to sensibly match a small ligand to a larger one will produce situations where an observer point will be quite far from the subject molecule but exactly $\gamma$ from the target.

In such a case, for the Coulombic comparisons in particular, a degenerate “similarity” may occur where a part of the target ligand that lacks charge will have close-by observers that are *far* from the subject molecule. In this situation, the nominal Coulombic feature values will both be close to zero, whether or not the subject molecule is charged proximal to the observer. This would result in some positive similarity where none exists in any real sense. Consequently, an overall weighting for each observer point is defined, which depends only on the subject ligand:

13 $$\begin{aligned} W_i&= g(stc_i^s - \gamma ,2.0) \end{aligned}$$

In order for a subject ligand to have a maximal score against a target ligand, it must match its surface shape, effective Coulombic field, and the positions and directions of its donor and acceptor atoms. Though not specified in Eq. 1, each of the individual similarity types (steric, Coulombic, and donor/acceptor) can be weighted by a constant (by default, all receive an equal weight of 1.0).

Ligand strain is measured by the nominal energy of the subject molecule above its global minimum (estimated during ligand preparation), multiplied by a negative constant (the default value is 0.05). The constants $\gamma , \sigma _{stc}, \sigma _{coul}, \sigma _{da}, \sigma _{da\theta },$ and $\alpha _{da}$ are, respectively: 4.0, 2.0, 8.0, 1.0, 0.5, and 0.25. These have not been optimized heavily; rather, values have been chosen such that the relative similarity of examples such as the one in Fig. 1 show intuitive values and relative ranks.

### Observer point placement

An alignment target may be a single molecule or it may be a set of mutually aligned molecules. An initial set of observer points is placed by first considering the union of all constituent atoms of the set. A sphere is placed with its center at the centroid, with its radius being 8.0 Å beyond the furthest atomic surface point. This sphere is tessellated such that the inter-point distance close to 1.0 Å. Each point is then moved as follows: (1) the minimum distance to the atoms is calculated; (2) the point is moved toward from the centroid in order to make the minimum surface distance $\gamma$ (4.0 Å). So, if the initial minimum distance is 10.0 Å, the point will be moved 6.0 Å toward the centroid); and (3) the process is repeated a total of ten times.

This procedure very rapidly converges on a set of points, each of which is nearly exactly 4.0 Å from the nearest atomic surface of the union of the alignment target molecules. Then, this dense set of points is pruned such that no pair of observer points is closer than 2.0 Å from one another (for geometric accuracy) or 4.0 Å from one another (for screening). The pruning procedure is iterative, building a final set of feature points from an initial arbitrarily chosen singlet. The procedure repeatedly identifies the unselected feature point whose minimal distance to its nearest neighbor within the selected set is maximal. If that distance is greater than the desired inter-point distance, the point is added; otherwise the procedure stops without adding the last identified feature point.

Note, however, that given a mixture of small and large molecules, it may be that some of the smaller ones are “hidden” by the larger ones and therefore do not have an observation point nearby. So, the process is repeated, but rather than using the merged set of all training ligands, each training ligand is processed individually. If any of the ligand-specific observer points is greater than 2.0 Å away from the evolving set, it is added to the set.

This process guarantees adequate sampling of the surfaces of all ligands that comprise an alignment target.

### Pose optimization

Subject ligands to be aligned to a particular target are prepared using ForceGen [25, 26], which provides an estimate of a ligand’s global energy minimum and also produces a diversity-optimized set of conformations within a fixed energy window (10.0 kcal/mol for non-macrocycles and 20.0 kcal/mol for macrocycles). The ForceGen sampling modes most relevant to this work are: *-pfast* with up to 50 conformers per molecule with limited ring search; *-pscreen* with up to 50 or 120 conformers per molecule depending on ligand flexibility (slightly deeper ring search); and *-pgeom* with up to 250 conformers per ensemble and thorough ring search.

The eSim method is controlled by similar options, corresponding to those that are used for conformational sampling: *-pfast*, *-pscreen*, and *-pgeom*. The *-pfast* mode downsamples ensembles to a maximum of 25 conformers each and treats conformers as being rigid, with limited fine-grained alignment optimization. The *-pscreen* mode downsamples ensembles to a maximum of 120 conformers each and also treats conformers as being rigid. It makes use of more extensive fine-grained alignment optimization for each subject ligand. The *-pgeom* mode downsamples ensembles to a maximum of 250 conformers each, and it treats conformers as being flexible during fine-grained pose optimization. Both the alignment parameters and rotatable ligand bonds are optimized for each subject ligand (using internal coordinates without varying bond lengths or angles).

In the first two modes, the conformational energy of the subject molecule’s conformers does not change, but in the geometric mode, the MMFF94sf force field is evaluated in order to produce accurate estimates of ligand strain during ligand configurational changes. Though results will not be discussed here, the eSim method also implemented a *-pquant* mode, which does no conformational downsampling and varies ligand pose using full Cartesian movement, allowing for more subtle conformational changes than are available using internal coordinates alone.

For each conformer of a subject ligand, a “shrink-wrapped” observer point set is created. This employs the same $\gamma$ value as in the construction of the target feature set, but no pruning of the observer points is done (the process can be time-consuming). As with the antedecent morphological similarity method [16], matching triangles are identified between the subject ligand’s conformers and a target ligand. Alignment transforms are calculated and applied to the conformers. The best scoring of the aligned conformers (according to the target ligand’s feature set) are retained. Those poses are then locally optimized, either using only the alignment parameters, alignment parameters and conformational internal coordinates, or using full all-atom Cartesian optimization.

Optimization is carried out using a quasi-Newton procedure, the Broyden–Fletcher–Goldfarb–Shanno algorithm (BFGS), which takes advantage of the analytical first derivative that is available because of the eSim function definition. Termination criteria for optimization are specific to each mode, trading off speed against more thorough optimization of the pose-dependent eSim value.

### Molecular data sets

Results will be presented for eSim virtual screening performance on the DUD-E set. While it has not been designed as a benchmark for ligand-based methods, it has become a de facto standard. Here, in addition to making use of the data as curated for comparison purposes, we augmented the set using additional data from the RCSB PDB.

#### Standard DUD-E benchmark

The set consists of 102 protein targets, provided as a single standard download. For each target, there is a receptor structure (PDB format), a bound ligand structure (SYBYL format), a set of known actives (SMILES format), and a set of computationally generated decoys (roughly 50 decoys per active compound, also in SMILES format). Data set preparation, for each target, was as follows:

1. *Cognate ligand* The cognate ligand, the target of similarity calculations, was used unmodified, with partial charges calculated using the ForceGen internal method [25]:
  sf-tools charge crystal_ligand.mol2 xtal
  –> xtal-orig-charged.mol2
2. *Initial Structure 3D Generation* The sets of curated active ligands and computationally generated decoys (actives_final.ism, decoys_final.ism) were re-formatted so that the first column contained SMILES and the second contained molecule identifiers. Structure generation, with automatic protonation for physiological pH (and partial charge calculation), was done as follows:
  sf-tools +reprot fgen3d actives.smi actives
  –> actives.mol2
  The analogous procedure was carried out for the DUD-E decoys (decoys.ism).
3. *Conformer generation* Conformer ensembles were generated using two levels of search depth: -pfast and -pscreen. Actives and decoys were combined into a single archive, and the procedure was as follows:
  sf-tools -pfast forcegen all.mol2 mols_fast
  –> mols_fast.sfdb
  The .sfdb file format is a binary compressed format to enable rapid reading of molecular ensembles, including different conformers with their corresponding energies. The procedure was repeated for the -pscreen option.

On average, the success rate for 3D conversion and conformer generation for the actives was 99.5%, and for the inactives, it was 98.4%. The small fraction of failures occurred in the generation of 3D structures due to a lack of valid MMFF94sf parameters or due to uninterpretable aromatic bond groups. All molecules for which 3D structures were produced had their respective conformer ensembles generated successfully.

#### Augmented DUD-E data set: DUD-E$^+$

In order to test the effect of target ligand choice and to comprehensively assess pose prediction, an augmented DUD-E set was constructed using fully automated procedures. Further details about the PSIM protein pocket similarity method and the automated curation protocol can be found in reports focused on binding site similarity and non-cognate ligand docking [27–29]. For each target, the following procedure was carried out:

1. *Alternate PDB Code Identification* The UniProt identifier associated with the DUD-E cognate protein structure was used to obtain a list of associated PDB codes. For human pathogens like HIV-1, HCV, Helicobacter pylori, Influenza virus, and Mycobacterium, the identical protein may have multiple UniProt IDs. In these cases, a representative UniProt accession code was used to obtain the largest set of variant structures. For example, P03742 was used for the nram target (influenza virus neuraminidase).
2. *Automated processing of PDB structures* For each target, the set of PDB codes was processed, as follows:
  sf-dock getpdb PDBList grind
  source grind-script

The scripts run a procedure for each PDB code, as follows:

1. The PDB biological assembly is downloaded using wget.
2. The sf-dock grindpdb command is used to heuristically infer components (protein, water, cofactors, and ligands), bond orders, and protonation/tautomer states. SYBYL mol2 files are produced for all components.
3. Quality measurements are calculated:
  - *Ligand strain by movement* A ligand is minimized under a quadratic positional constraint on its heavy atoms. It is retained if RMSD from the original coordinates does not exceed 0.55 Å. The ligand is then freed from positional restraint.
  - *Ligand strain by energy* The ligand’s pose is optimized in Cartesian space with both the internal MMFF94sf force field and the Surflex-Dock scoring function. Its MMFF94sf energy is calculated in this protein-bound locally optimal state. The ligand is then minimized outside of the protein. The gap in MMFF94sf energy values for these two conformations is considered in the context of the number of non-hydrogen atoms of the ligand. If the difference between the two energy values does not exceed 0.50 kcal/mol/atom, the ligand is retained.
  - *Structure quality by movement* If the RMSD between the experimental coordinates and the optimal scoring pose from local optimization is less than 1.25 Å, the ligand is retained.
  - *Structure quality by ligand efficiency* The optimal docking score (Surflex-Dock scoring function, nominally in units of $\mathrm{pK}_d$) divided by the number heavy atoms is at least 0.10 $\mathrm{pK}_d/\mathrm{atom}$, the ligand is retained.
  - *Structure match to alternate curation* Graph matching is done between the final ligand and the corresponding SMILES-based molecular structure (and tautomeric variants) from the RCSB Ligand Expo. If there is a match, the ligand is retained.

The overall procedure made use of 9018 PDB structures across the 102 DUD-E targets. From this automatic procedure with relatively stringent quality assessment, 5459 PDB codes resulted in at least one small-molecule (non-cofactor) ligand whose structure passed the series of quality tests just described.

For each target, these liganded protein binding sites were mutually aligned and five representative variants were selected based purely on the basis of local protein binding site similarity. In general, the pocket alignment procedure will produce multiple independent alignment trees, each with variants of a different binding site. These are ordered from the most populated to the least. Here, we considered the three most populated alignment trees, choosing binding site exemplars from within each, as follows: 
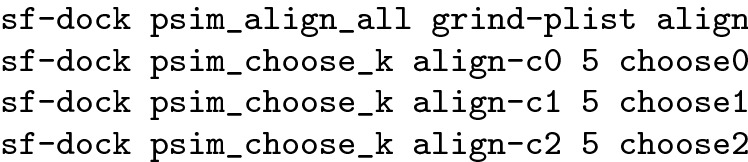


In nearly all cases, the largest cluster of binding sites (labeled “c0”) was the site that matched the DUD-E binding site. In just 14 cases, the second-largest cluster matched (“c1”), and in a single case the third cluster matched (“c2”). Cluster identification was done manually in cases where the exact PDB ligand from the DUD-E set did not appear in the automatically generated aligned binding site clusters. For 92 targets, at least five variants were identified in this manner, with a total of 5651 ligand-containing binding sites.

Note that for proteins whose biological assembly is multimeric, the same ligand (possibly in slightly different conformations) may be present more than once from within the *same* structure. The ten DUD-E targets with less than five variants were: ampc, cp2c9, cp3a4, cxcr4, drd3, igf1r, ital, kit, kith, and kpcb. Those targets were not used in analyses of target ligand variation or pose prediction.

For the 92 targets with at least five variants, the mutually aligned ligands corresponding to the selected representative protein variants were collected into a single file (xtal-alt.mol2). Also, the set of ligands for the mutually aligned full set of variants was collected into a single file (xtal-all-ligs.mol2). Using the definitions discussed in the Introduction, the total number of tractable non-self alignments (with molecular overlap $\ge 0.8$*or* direct atomic overlap $\ge 0.5$) was 381,467. The most challenging subset (MO $\ge 0.8$*and* DAO $< 0.5$) contained 185,308 examples (48.6%). The least challenging subset (MO $\ge 0.8$*and* DAO $\ge 0.5$) contained 170,550 examples (44.7%). The remainder (MO $< 0.8$*and* DAO $\ge 0.5$) contained just 25,609 examples (6.7%).

**Table 1:** Summary of performance of ten ligand similarity methods on the full DUD-E set of 102 targets along with a molecular indexing control (SF-imprint)

Method	Mean	% AUC	% AUC	% AUC	% AUC	% AUC	% AUC	Time
	ROC area	< 0.50	$\ge$ 0.60	$\ge$ 0.70	$\ge$ 0.80	$\ge$ 0.90	$\ge$ 0.95	Mols/s
eSim -pscreen	**0.755**	**5**	**81**	**69**	**43**	**17**	**8**	12.3
eSim -pfast	**0.736**	**9**	**82**	**62**	**34**	**14**	**5**	61.2
eSim -pfastf	0.706	**5**	79	53	26	6	3	274.9
ROCS	0.663	18	66	44	21	9	3	50
ROCS (shape)	0.596	31	54	25	12	1	0	50
SF-Imprint	0.570	36	37	19	7	1	0	> **200,000**
WEGA	0.564	31	44	19	6	0	0	26.7
OptiPharm (robust)	0.560	32	41	15	5	0	0	12.0
VAMS	0.560	36	40	14	3	0	0	5000
USR	0.554	35	43	20	5	1	0	6000
USR (shape)	0.520	43	28	13	2	0	0	6000

Standard deviation of the mean ROC areas was 0.13–0.16. The top two performance values in each column are bolded. Performance improvements of all three eSim modes over all other methods was highly statistically significant (see text for details). Performance data for ROCS, USR, and VAMS were taken from [19] (times are approximate, based on 25 conformers per molecule), and data for OptiPharm and WEGA were taken from [20]

### Computational procedures and statistical analysis

The results reported here were generated using Surflex Platform version 4.440. Ligand preparation for the various analyses has been discussed above. For the screening assessments compared with alternate methods, the following procedure was used: 
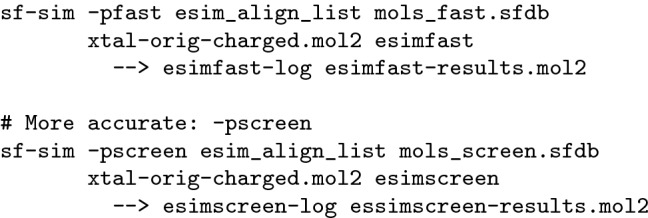


ROC areas were computed in the standard fashion [2, 3, 9]. For the assessment of pose prediction, the following commands were wrapped in a procedure that also calculated topological similarity, molecular overlap, direct atomic overlap, and RMSD results for the subject and target molecules: 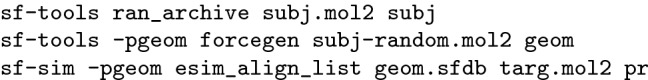


RMSD was calculated using all heavy atoms, with consideration of equivalent subgraph isomorphisms for the subject molecule in its bound pose and predicted poses.

For ROCS-color, pose prediction performance was assessed on the DUD-E$^+$ benchmark. Initial conformations were the same randomized versions as used for eSim. Conformational search was carried out using OMEGA, with default parameters, except that the -maxconfs parameter was increased from 200 to 250 to provide for equivalent depth in sampling. ROCS was run using its default behavior, which ranks final predicted poses using ROCS-color approach. The top-ranked 20 poses were retained for calculation of symmetry-corrected RMSD against the bound pose of each subject ligand, exactly as with eSim. The following commands were employed (the OMEGA macrocycle variant was run only on those molecules identified as macrocycles by omega2): 
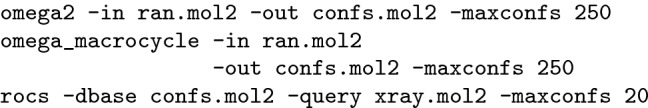


For this work, OMEGA Version 3.1.0.3 [30] and ROCS Version 3.3.0.3 [22] were used (OpenEye Scientific Software, Santa Fe, NM, USA).

For the statistical comparisons to other methods, because performance data were available on a per-target basis for the full 102-target DUD-E set [19, 20], paired t-tests were used in order to provide statistical power. Normality of the distributions of differences between ROC areas was checked in order to ensure applicability of the paired sample t-test (using the Kolmogorov-Smirnov test of normality).

Calculations for eSim were done on a workstation equipped with dual Intel Xeon Gold 6154 CPUs, operating at 3.00 GHz, with a total of 36 physical computing cores, each capable of running 2 threads. All timings were the result of single-core, single-thread computations except for the single example of the macrocycle discussed in reference to Fig. 9, which was run using 25 threads. Calculations for pose prediction assessment of ROCS were done on a computing server with 4 Intel Xeon Gold 6140 CPUs, operating at 2.3 GHz, with a total of 72 computing cores. ROCS was run as single-core, single-threaded computations.

Additional details about the data sets, computational procedures, and about software availability are available at www.jainlab.org.

## Results and discussion

Three different performance analyses were done to characterize eSim with respect to virtual screening utility and pose prediction. The first was a direct, apples-to-apples comparison with seven other similarity approaches on the full DUD-E set of 102 targets, using the given crystallographic ligand as the query in each case. The second explored the use of alternate ligands (singly and as aligned sets) as queries. The third analysis was a comprehensive characterization of pose prediction.

### DUD-E: full target set comparison

Table 1 summarizes the virtual screening performance for three eSim modes and five alternate methods (two of which were run with shape-only and shape plus “color” variations [19]). A 3D molecular indexing approach is also included as a baseline. The first column shows mean ROC area (AUC), where 0.5 indicates random performance and 1.0 indicates perfect separation of curated active ligands from the DUD-E computationally generated decoys. The second column shows the percentage of targets for which each method performed nominally worse than random. The next five columns show the percentage of cases with AUC of at least 0.60–0.95, respectively. The final column characterizes computational speed in molecules per second, with all methods run using single-core, single-thread procedures. In all columns, the best two performance values are shown in bold face.

The eSim *-pscreen* method performed statistically significantly better than all other methods and variations. P-values (paired t-test of AUC values, two-tailed) were extremely low (p less than 5$\times 10^{-20}$) compared with ROCS (shape), WEGA, OptiPharm, VAMS, USR, and USR (shape). Compared with ROCS (color), eSim *-pscreen* had a p value of 8$\times 10^{-13}$. Both of the faster eSim modes (*-pfast* and *-pfastf*) behaved similarly, with extreme p-values compared with all non-ROCS-color approaches (less than $10^{-13}$ for both variants in all cases). Compared with ROCS-color, we also observed highly significant p-values for the faster screening modes (less than $10^{-9}$ and $10^{-3}$, respectively).

As discussed earlier, paired t-tests are appropriate due to the near normality of the distributions of differences in paired AUC values between methods, and they provide much better statistical power than distribution-level tests of performance differences. Note that prior reports on virtual screening performance using the DUD-E set made use of relatively low power tests, which fail to distinguish mean AUC performance differences even as large as 0.04 across the 102 targets [19]. The performance gains in terms of AUC for eSim were large enough that even unpaired t-tests yielded statistical significance in all comparisons of eSim variations with alternate method variations.

**Fig. 5:**
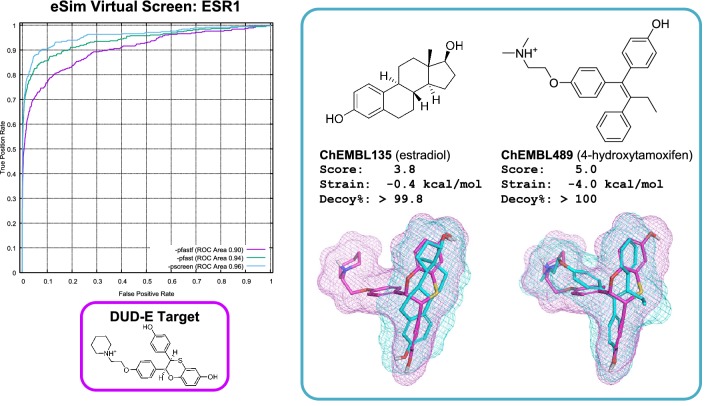
Virtual screening performance for eSim in three screening modes (*-pfastf*, *-pfast*, and *-pscreen*, upper left plot), along with two examples of positive ligands, their top-scoring poses (cyan), scores, strain values, and the percentage of decoy molecules whose scores were exceeded

Except for eSim (all variants) and ROCS (both variants), the remaining similarity methods did not substantially exceed random performance (AUC of at least 0.60) in more than half of the cases. Further, in no cases did those methods produce exceptional performance (AUC at least 0.95) and only in a single instance did they collectively produce very good performance (AUC at least 0.90). While the USR and VAMS methods were nominally fast (thousands of molecules per second, ignoring molecular I/O time [19]), the WEGA and OptiPharm methods performed only slightly better, yet they are two orders of magnitude slower.

It is important to note that the mature and extremely fast molecular indexing approach implemented within Surflex-Sim [15, 31, 32] performed marginally better than than all of the non-ROCS alternative methods, and it was *many times* faster than even the VAMS and USR methods. The approach constructs a 20-dimensional vector for each molecule (called an “imprint”), where each vector value is the maximal similarity of the ligand to a particular basis molecule. These vectors are used to infer relative similarities using Euclidean distance. The calculation is disk I/O bound, processing over 200,000 molecules per second, and could be made significantly faster by employing a binary file format for the imprints. The focus of this paper is on fast and high-quality similarity calculations, though, not on ultra-fast calculations with more moderate expected enrichment performance. None of the methods with mean AUC from Table 1 that were less than that obtained by the simple molecular indexing approach will be discussed further.

Note that, as standard practice, we have traditionally employed 2D similarity methods as essential controls in assessing the performance of 3D molecular similarity [4, 33]. However, in the case of the DUD-E set, because the decoys were generated *specifically* to have low 2D similarity to the curated actives, the set is therefore inappropriate for use in assessing 2D methods, as pointed out by its developers, Mysinger et al. [18].

Figure 5 shows the ESR1 (estrogen receptor alpha) example from the DUD-E set, which consists of 383 curated actives, 20,685 decoys, and makes use of the ligand of PDB structure 1SJ0 as a similarity target. This particular DUD-E case illustrates some of the critical challenges for similarity methods. First, the target ligand may or may not be representative of the majority of actives, here showing significant differences in size and formal charge from the two typical active ligands depicted. Recall that the DUD-E active site exemplar was chosen specifically for suitability within a docking benchmark, and the particular cognate ligand is quite arbitrary from a ligand similarity perspective. Second, there may or may not be significant atomic center overlap between the subject molecule and target molecule (in their bound poses), which may pose problems for both alignment optimization and scoring, depending on the particular method involved.

ESim performed very well on ESR1 (ROC areas of 0.90, 0.94, and 0.96 for the *-pfastf*, *-pfast*, and *-pscreen* modes). The average for the alternate methods was 0.63 with a maximum of 0.73 (ROCS color). The eSim score of 3.8 for estradiol placed it above 99.8% of the scores for the decoys, and 4-hydroxy tamoxifen scored higher than all of the decoys. In both cases, the predicted poses were reasonable in terms of overall correspondence with the target ligand.

**Fig. 6:**
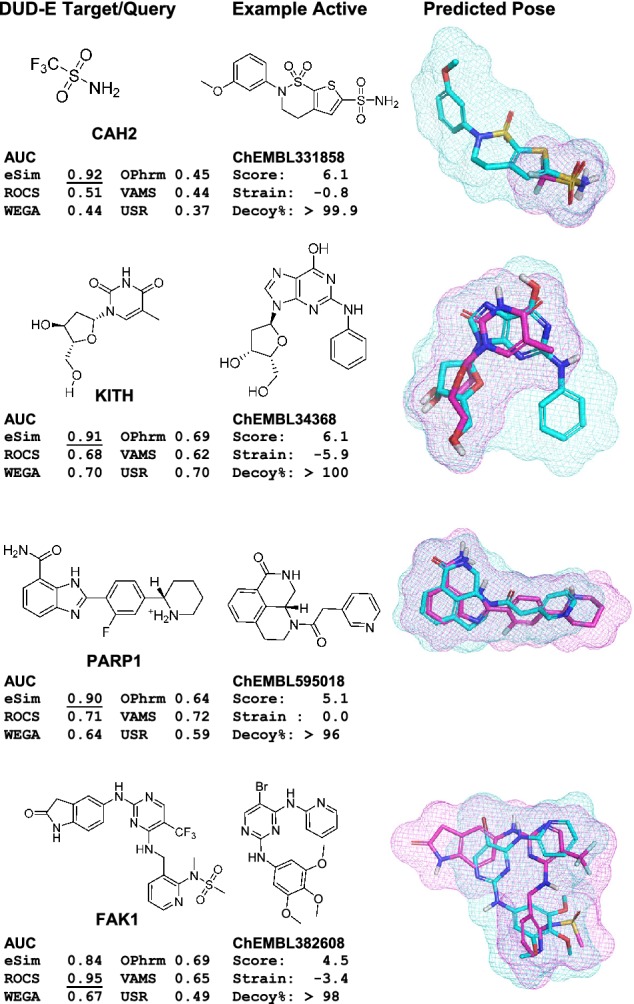
Examples typical of eSim performance compared with alternative approaches

Figure 6 shows an additional four specific target examples from the DUD-E set. In the first three cases (carbonic anhydrase 2, thymidine kinase (KITH), and poly-ADP-ribose polymerase 1), eSim showed significant advantages over the other approaches, with ROCS (color) showing either the best performance among alternative methods or very close to the best. The remaining target (focal adhesion kinase 1) was atypical (see Table 2), where eSim performed significantly worse than the best alternate method (ROCS).

Carbonic anhydrase presented a difficult challenge for methods that were overly dependent on congruent volumes because the query ligand was small relative to the typical active to be retrieved (roughly a third of the size based on Van der Waals volume). However, the electronic “signature” of the particular query molecule was sufficient for eSim to obtain excellent retrieval. Note that the predicted alignment of the large active to the small query aligned the common “warhead” well (the sulfonamide interacts with a catalytic zinc cation in the protein active site).

Volumetric discrepancy, while probably contributing to the large performance differences observed, did not explain performance differences overall. Numerous cases existed where query volume and average active volume were close to identical (e.g., ESR1), but where eSim showed similar performance advantages. Thymidine kinase was a case where relatively consistent performance was obtained from the non-eSim approaches (roughly 0.70 AUC). In that case, the query ligand was slightly smaller, on average, than the actives (about two-thirds the size). In the case of PARP1, the query ligand was, on average, 6% smaller by volume than the actives, but eSim still maintained a substantial advantage. The target FAK1 was one of just two targets where ROCS performed slightly better than eSim (AUC better by 0.10 or more), and the volumetric difference between query and actives was similar to that of PARP1 (the query was, on average, just 6% larger than the actives).

#### Size disparities in similarity calculations

The issue of size disparity between query and actives is an important one. The bias of many approaches is to attempt to make similarity values symmetric, such that in order to reach maximal values, the subject ligand must cover the query *and* vice-versa. However, within the DUD-E set alone, the average relative volume of the query ligand compared with the curated actives ranged from 0.3 (carbonic anhydrase) to 1.7 (CYP3a4). In many cases, the standard deviation of the volumes of the actives approached the magnitude of the mean, exhibiting a large variety of sizes.

Further, as a purely physical matter, in all cases of ligand binding, there must be some pathway from solvent to binding site, and in many cases, this pathway can remain at least partially open during the binding event. For cases such as carbonic anhydrase, the ligand size variation is in the direction of solvent exposure, which is typical. The eSim approach of scoping the similarity calculation based on the query molecule and measuring the extent that a new ligand can “cover” the query appears to be well justified.

Note that similarity methods including ROCS allow for the use of either Tanimoto (the default) or Tversky weighting in similarity calculations. The former approach is symmetric in the treatment of query and subject ligand, and the latter is weighted in favor of the query in the typical application. While there is no general agreement on the superiority of one over the other, the Tversky approach has been shown to produce some improvements in screening performance [34, 35]. However, to our knowledge, this issue has not been explored on the full DUD-E benchmark, though marginal improvements have been shown on a subset [35].

It seems clear, though, that symmetrical treatment of query and subject molecules is not supported by the observed size diversity among actives, the physical process of ligand binding, nor the performance data presented here.

**Table 2:** Performance of eSim (*-pscreen*) compared with the maximum AUC over the alternate methods for the 102 DUD-E targets

Target	eSim	Max (other)	Target	eSim	Max (other)	Target	eSim	Max (other)
aa2ar	0.76	0.71	fabp4	0.83	0.80	mmp13	0.72	0.69
abl1	**0.73**	0.61	fak1	0.84	**0.95**	mp2k1	**0.63**	0.53
ace	0.75	0.75	fgfr1	**0.71**	0.56	nos1	0.53	0.44
aces	0.49	0.52	fkb1a	0.60	**0.72**	nram	0.90	0.87
ada	0.91	0.89	fnta	0.68	**0.78**	pa2ga	0.74	0.71
ada17	**0.80**	0.64	fpps	0.95	0.99	parp1	**0.90**	0.72
adrb1	**0.70**	0.47	gcr	0.64	0.63	pde5a	**0.73**	0.62
adrb2	**0.65**	0.48	glcm	**0.78**	0.52	pgh1	0.57	**0.73**
akt1	**0.58**	0.41	gria2	0.60	0.68	pgh2	0.84	0.81
akt2	**0.66**	0.41	grik1	0.70	0.73	plk1	**0.82**	0.56
aldr	0.71	0.69	hdac2	0.53	0.45	pnph	0.88	0.92
ampc	0.62	**0.76**	hdac8	0.85	0.77	ppara	0.90	0.86
andr	0.71	0.69	hivint	0.49	0.47	ppard	**0.81**	0.69
aofb	0.46	0.44	hivpr	0.84	0.78	pparg	0.79	0.73
bace1	0.53	0.54	hivrt	0.71	0.69	prgr	0.81	0.72
braf	**0.77**	0.64	hmdh	0.82	0.91	ptn1	**0.57**	0.33
cah2	**0.92**	0.51	hs90a	0.80	0.73	pur2	**1.00**	0.90
casp3	0.55	0.58	hxk4	0.90	0.81	pygm	0.46	**0.58**
cdk2	**0.80**	0.72	igf1r	0.61	0.55	pyrd	0.83	0.90
comt	0.99	0.90	inha	0.68	0.72	reni	0.79	0.73
cp2c9	0.52	0.46	ital	**0.77**	0.58	rock1	**0.79**	0.61
cp3a4	0.58	0.55	jak2	0.74	0.81	rxra	0.93	0.90
csf1r	0.80	0.66	kif11	0.73	**0.83**	sahh	1.00	1.00
cxcr4	0.79	0.78	kit	**0.69**	0.48	src	0.67	0.67
def	0.85	0.86	kith	**0.91**	0.75	tgfr1	**0.84**	0.72
dhi1	0.59	0.68	kpcb	0.85	0.80	thb	0.87	0.89
dpp4	0.73	0.68	lck	0.55	0.46	thrb	**0.83**	0.64
drd3	**0.46**	0.33	lkha4	0.84	0.79	try1	**0.87**	0.73
dyr	**0.95**	0.74	mapk 2	0.86	0.86	tryb1	**0.83**	0.48
egfr	0.75	0.77	mcr	0.72	0.79	tysy	**0.92**	0.79
esr1	**0.96**	0.73	met	0.87	0.81	urok	**0.81**	0.50
esr2	**0.97**	0.82	mk01	0.79	0.73	vgfr2	0.75	0.72
fa10	0.73	0.71	mk10	0.56	0.56	wee1	1.00	0.98
fa7	**0.96**	0.86	mk14	0.58	0.61	xiap	0.94	0.92

Differences of 0.10 or greater are bolded

#### Summary of full DUD-E performance

Table 2 shows the performance of eSim (*-pscreen*) compared with the *maximum* AUC (for each target) over all seven alternate methods discussed above. In addition, one molecular indexing approach is included as a baseline reference [32]. Overall, the mean of the per-target maximum AUC for the alternate methods was 0.692, compared with 0.755 for eSim (p $< 10^{-7}$). There were 33 cases where eSim performed better by at least 0.10 AUC units, compared with just 7 for the converse. There were 12 cases where eSim’s advantage was 0.20 or greater, with none in the converse direction. This comparison represents the best possible case for the set of alternate methods: cherry-picking from among them. In this best-case scenario, one must be able to, *a priori*, perfectly guess which method will perform best for each of the 102 DUD-E targets. Even in that case, eSim performed consistently better.

There were three cases (pur2, sahh, and wee1) where eSim (*-pscreen*) produced perfect separation between actives and decoys. All three of these cases exhibited very high or perfect performance for at least one of the alternative methods. Each contained active ligands with an extremely distinctive motif. These motifs were apparently eliminated from the DUD-E decoy sets, both in 2D and 3D form, by the procedures designed to reduce the presence of true ligands among the computationally generated decoys.

In terms of speed, there are three levels of accuracy/speed parameterizations for eSim virtual screening: *-pscreen*, *-pfast*, and *-pfastf*. All three modes performed statistically significantly better than all alternative methods. The *-pfast* mode performed nearly as well as the *-pscreen* mode (mean AUC values of 0.74 and 0.76, respectively). On a single computing core, eSim *-pfast* processes over 60 molecules per second, which amounts to over 5,000,000 molecules per day. For most applications, especially using either multi-core workstations or multi-node cloud-based computing instances, this speed is sufficient. It is also faster than ROCS, WEGA, and OptiPharm (though the latter two methods produced near-random performance on a majority of DUD-E targets).

The VAMS and USR methods were nominally very fast, but they showed the weakest performance on the DUD-E set. They do not outperform 3D molecular indexing, a general method which has been known for over twenty years [15, 31, 32, 36], in terms of either quality or speed. The eSim *-pfastf* mode is substantially faster than the other eSim modes, while maintaining statistically significant performance advantages (mean AUC of 0.71 at 275 molecules per second). On a modest 100-core computing cluster, throughput of over 2 billion molecules per day is possible, which is roughly half the size of currently-available make-on-demand molecular libraries [37].

Of note, the ROCS approach has a GPU-based approximate implementation that is faster than the VAMS and USR approaches [38], though we are not aware of direct performance assessment of FastROCS on the DUD-E benchmark. In principle, the eSim approach is amenable to a GPU-based implementation, though it is not clear what the trade-off would be between speed and the necessary approximations for effective speed optimization. Using eSim (*-pfastf*), screening several billion molecules in less than a day would represent a modest cost on spot-priced cloud computing instances. This would not require special-purpose hardware, and the trade-off in terms of accuracy is relatively modest.

The eSim method produced, by a meaningful margin, the strongest performance on the DUD-E set and was also the fastest among the remotely competitive alternatives where performance data were available. It is difficult to pinpoint reasons for the performance advantages observed. The eSim method shares very little in common with any of the other approaches, using surface-shape instead of volume, comparing full electrostatic fields, and considering the details of hydrogen-bonding preferences including directionality.

### eSim: contributions of terms

To help understand what drove eSim virtual screening performance, the method was run using different weightings on its core components. Recall that the eSim function has three primary contributions: pure surface shape information, electrostatic field comparison, and measurement of differences in donor/acceptor functionality. Equal weighting of these three contributions produced the results in Table 1.

**Table 3:** Performance of eSim (*-pscreen*) under different combinations of weighting for the primary similarity terms for the full 102-target DUD-E set, compared with Surflex-Sim (*-pscreen*), ROCS (with color), and ROCS (shape only)

	AUC	Shape	Coulombic	Don/Acc or Color
eSim				
All	**0.755**	1	1	1
Shape	0.620	1	0	0
Coul.	0.687	0	1	0
Don/Acc	0.736	0	0	1
S + C	0.672	1	1	0
S + DA	0.749	1	0	1
C + DA	**0.765**	0	1	1
SF-Sim	0.721	1	–	1
ROCS				
Color	0.663	1	–	1
Shape	0.596	1	–	0

Table 3 shows the effects of different combinations of weightings using the *-pscreen* mode, compared with Surflex-Sim, ROCS (color), and ROCS (shape only). Single-component similarity performed significantly worse in each case than the full eSim calculation. The eSim shape-only results were slightly better than the ROCS shape-only results, and they were also significantly better than all of the other alternative methods (see Table 1), whether they considered pure shape or aspects of ligand polarity. There is intuitive appeal to the idea that characterizing the congruence of molecular surface shapes would be more effective than reliance on atom-center congruence, which drives similarity in Gaussian-overlap methods [21, 39]. However, the modest advantage observed here should not be considered to be definitive.

What was more surprising was that the Coulombic-only results were slightly better than all of the alternative methods and that the hydrogen-bonding-only results were substantially better. Recall, however, from Eqs. 1 and 13 that the overall similarity contribution at each observer point is multiplicatively weighted by the extent to which the subject molecule in its particular pose is at the preferred distance from the observer to the query. This weighting has the effect that, for either the Coulombic or hydrogen-bonding comparisons to contribute meaningfully to similarity, there must be some degree of local shape congruence. The combination of shape and hydrogen-bonding similarity was nonetheless synergistic, but the combination of shape and Coulombic similarity was marginally worse than Coulombic similarity alone.

Most surprisingly, the combination of Coulombic and hydrogen-bonding similarity produced a mean AUC of 0.765, slightly better than the combination of all three components (p $= 0.001$ by paired t-test). Performance was very strongly correlated, with AUC values between the two variants having Pearson’s correlation of 0.98. At a finer level of detail, in looking at the alignments produced, the full equal-weighted eSim approach yielded “tighter” alignments that were more concordant with intuition. In a later section, pose prediction and this aspect of better alignment accuracy will be explored.

Also, recall that the individual parameters that control eSim’s Gaussian and sigmoidal response functions were not optimized with respect to DUD-E performance. It is likely that systematic exploration of these parameters will lead to improvements in both screening effectiveness and pose prediction accuracy.

The eSim method is closely related to the Surflex-Sim morphological similarity approach. The latter lacks a Coulombic field comparison component, and it has some differences in the calculation of directionality of hydrogen bonds as well as in the details of observer point weighting. The eSim approach, limited to the shape and hydrogen-bonding terms, is the most direct analog. Performance of the morphological similarity approach on its fastest setting (*-pscreen*) produced an average AUC of 0.721, which was, as expected, close to that observed with eSim (*-pscreen*) performance when limited to shape and hydrogen-bonding features (AUC of 0.749). It appears that the reformulated directionality and weighting schemes yielded some improvement. In addition, the Surflex-Sim approach in its fastest screening mode is somewhat slower than eSim in its *slowest* screening mode. The eSim approach is much faster for comparable overall performance (using the *-pfastf* setting, eSim is roughly 30–50 times faster), and it produces significantly better performance in its more thorough modes (which are still faster than Surflex-Sim).

Shape-only eSim showed similar performance to the best of the other shape-only method variants. The full calculation that included Coulombic and hydrogen-bonding components, however, was clearly better than other approaches. So, it appears that overall performance gain derives primarily from the new approach to comparing electrostatic fields coupled with the directional treatment of hydrogen bond comparisons.

### Effects of query ligand

It is worth remembering that the DUD-E benchmark was developed for assessing the performance of docking methods [18]. Over 3500 protein structural variants were tested in structure-based screening workflows using DOCK 3.6 in order to identify the variant most suitable for docking assessment. So, from the perspective of ligand-based modeling, the particular ligands used as virtual screening queries are essentially arbitrary. There is no reason to believe that they are ideal for ligand-based screening, just that their cognate binding pockets are good choices for docking.

**Table 4:** Performance of eSim on 92 targets for which alternate query ligands were automatically curated

Query	AUC	AUC	AUC
Ligand(s)	-pscreen	-pfast	-pfastf
DUD-E Original	0.763	0.743	0.711
Best single alternate	0.818	0.799	0.758
Joint 5 alternates	0.818	0.802	0.770

To assess the effects of query ligand, we curated over 9000 PDB structures, by searching using UniProt identifiers for the DUD-E targets. An automated procedure yielded, for 92 targets, between 5 and 359 variant ligands and associated aligned protein binding sites (see Methods for details). Five variants for each target were chosen using a k-means clustering algorithm based on local protein binding site similarity [28, 29]. The cognate ligands for these selected variants were used as alternative queries for eSim.

This was done in two ways. First, each of the five individual ligands was used singly as a query (as in the experiments above), exactly as the original DUD-E ligand was used. Second, the *set* of mutually aligned ligands was used as a *single* combined multi-molecule query. Table 4 summarizes the results. As expected, the best performing from among five arbitrarily chosen query ligands (second row), produced significantly better results than the single arbitrary original DUD-E ligand.

Rather than using each alternative query ligand separately, it is possible to use them simultaneously, without requiring any experimentation to determine which individual ligand might be best. Within the eSim formulation, this is done by taking the *maximum* similarity over the multiply-aligned ligands at each observer point (in Eq. 1, the maximum operation would be inside the operand of the summation). In this manner, a subject ligand “tries” to look like the best-matching parts of the amalgamated query ligand set.

**Fig. 7:**
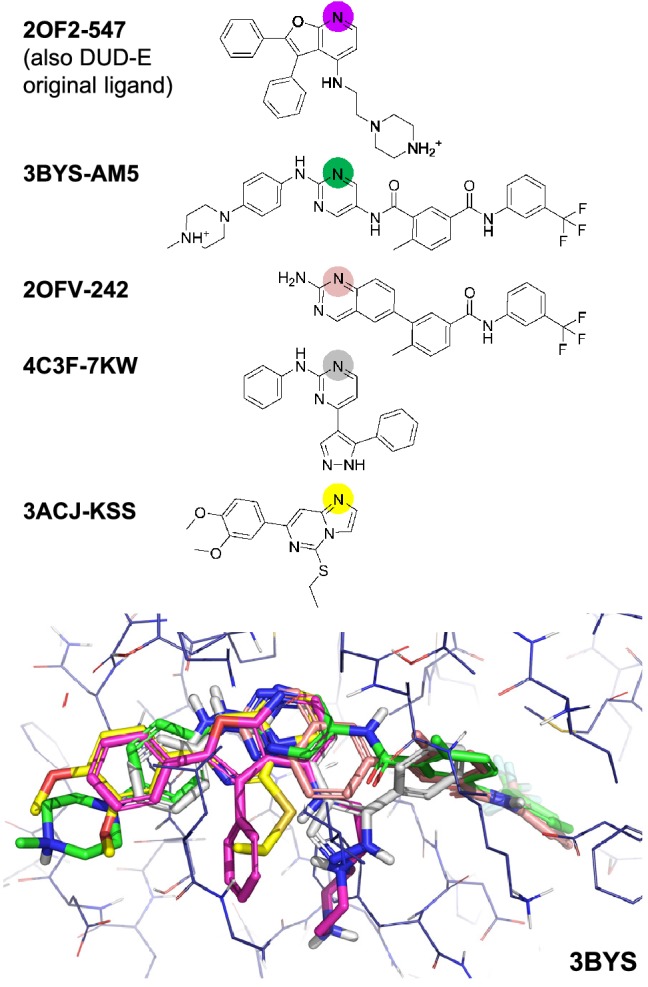
Alternate ligands for LCK, automatically curated, also included the original DUD-E ligand

Using the combination of five ligands, eSim achieved performance equivalent to (or marginally better than) that seen with the single best ligand in all three screening modes (bottom row of Table 4), but this procedure requires no *a priori* knowledge of additional ligand binding behavior. Figure 7 shows the LCK example, which obtained an improvement from 0.55 AUC (eSim *-pscreen*) to 0.86. The original DUD-E ligand was a uniformly poor representative regardless of similarity method (the maximum AUC for the non-eSim methods was 0.46).

All five ligands fill different parts of the active site, each sharing a hinge-region hydrogen bond (the corresponding nitrogen atoms are indicated with colored circles for each 2D depiction). Not surprisingly, the single best query ligand was 3BYS-AM5, which spanned the full width of the site. Using 3BYS-AM5 by itself, the eSim AUC was 0.81, slightly lower than what was seen with the mutual overlay of the five ligands shown. That single ligand does not fill the bottom of the pocket, and the joint overlay appears to provide additional information.

Making use of five alternates as a joint query requires roughly three- to four-fold the amount of computational time than using a single query ligand. Some efficiency beyond a linear cost is gained by sharing the burden of certain search operations instead of bearing them independently.

It is fairly common for structure-enabled discovery projects to have multiple crystal structures of diverse chemical scaffolds. As an adjunct to virtual screening with docking approaches, using the joint overlay of ligands that span an active site in a ligand-based screen should provide valuable orthogonal results, without requiring especially careful choice of the particular ligands.

### Ligand-based pose prediction

Of course, it is also quite common for projects to lack protein structure information, in which case, use of multiple-ligand queries depends on generating a joint alignment, a process that relies on the extent to which the poses that maximize a similarity function reflect what is observed experimentally.

There are three basic forms of the ligand-based pose prediction problem. The hardest form takes N ligands, each with partial overlap to at least one other ligand in the bound state, and builds a full alignment clique beginning from an agnostic starting point. There may be pairs of ligands within a clique that do not overlap at all. This version of the problem is seen in 3D QSAR, and it is, in general, extremely challenging. An easier form of the problem, which must be reasonably well-solved in order to tackle the hardest form, takes a pair of ligands, again with some degree of partial overlap, and produces a set of mutual alignments from an agnostic starting point. The easiest form of the problem simplifies the task by making use of the crystallographic pose of one ligand of a pair, here called the target ligand.

**Fig. 8:**
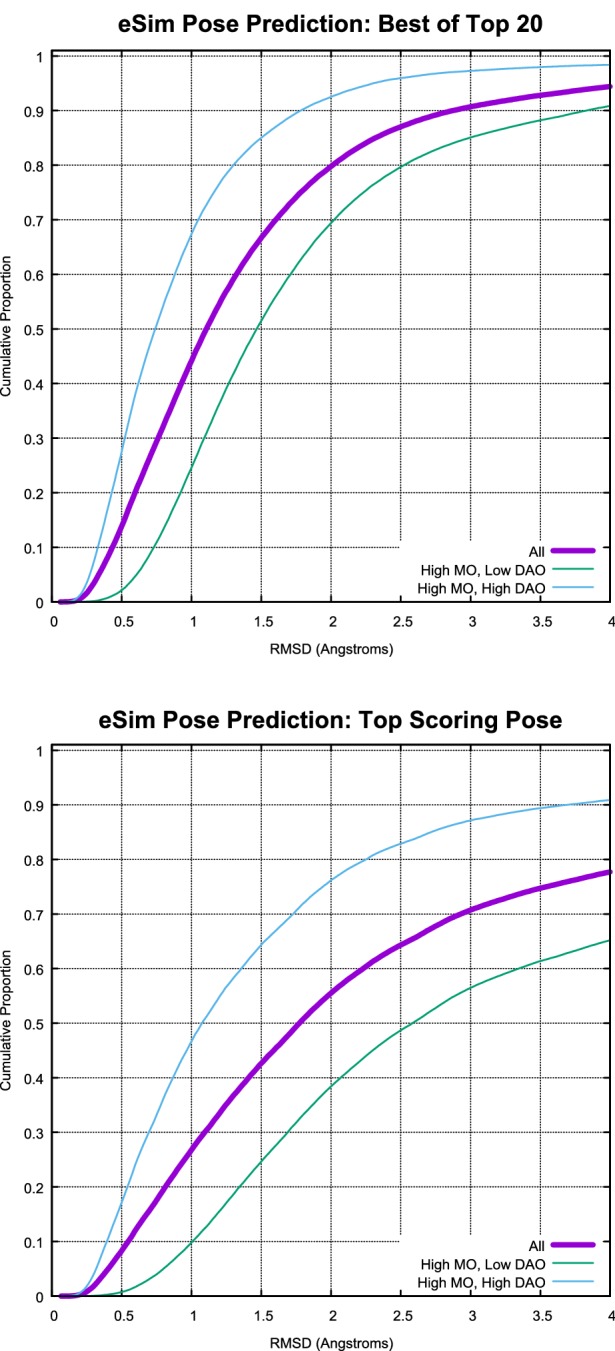
Plots of best predicted pose (top) and top scoring pose (bottom) for eSim (*-pgeom*), showing the cumulative histograms for all 381,467 cases (purple), and the roughly equal-sized subsets with low DAO (green) and high DAO (cyan)

The extensive curation of liganded binding site variants for the DUD-E set forms a very large and pharmaceutically relevant benchmark for assessing performance for all three versions of the ligand alignment problem. Here, we will address the simplest of the three forms. As described in the Introduction, we have identified 381,467 ligand alignment pairs that have the following properties: (1) they bind the same active site in known configurations; (2) the subject molecule and target molecule are non-identical; and (3) either the subject molecule has at least 80% molecular overlap (MO) to the target *or* it has at least 50% direct atomic overlap. Note that this allows for the case where a small subject ligand is to be aligned to a much larger target ligand, creating significant uncertainty about even the gross location of its bound pose. Note that any non-overlapping portions of a subject molecule have no constraint to guide pose choice except for molecular energetics.

For testing eSim pose prediction accuracy, the subject ligands were fully randomized to eliminate memory of their bound configurations, then conformationally sampled using the ForceGen *-pgeom* level of search. The eSim alignments were carried out using the *-pgeom* eSim mode as well.

**Fig. 9:**
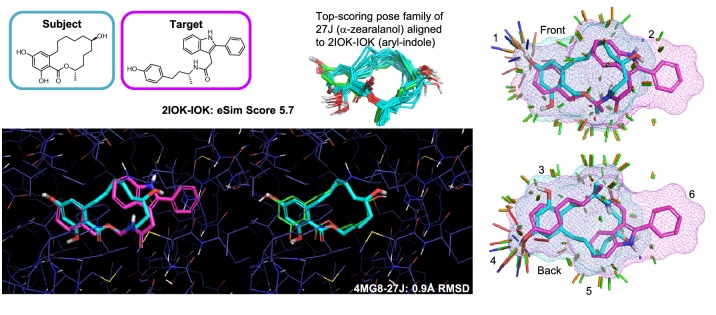
Macrocyclic ligand of estrogen receptor alpha shown in its best predicted pose (cyan), beginning from a memory-free starting point, using eSim. *pgeom* with the ligand of 2IOK as the alignment target (magenta); the RMS deviation from the bound pose (green) was 0.9 Å; at right, similarity “sticks” are shown for front and back views of the alignment, indicating shape similarity (green), compatible donors (blue), compatible acceptors (red), and portions of the molecules where the electrostatic fields are similar (orange)

Figure 8 shows the cumulative histograms of RMS deviation for best pose among the top 20 returned (top) and for the top scoring pose (bottom) for all cases and broken into the two largest subsets from the pie-chart shown in Fig. 4. Overall, 80% of the cases produced a pose within the top 20 returned with RMSD $\le$ 2.0 Å. The subset with high direct atomic overlap was clearly easier for pose prediction, with 93% meeting the 2.0 Å threshold. The subset with low direct atomic overlap, which includes many pairs of molecules with dissimilar scaffolds, yielded just 70% success at 2.0 Å RMSD. The same pattern was observed with respect to top-scoring pose, but with a nearly 40 percentage point gap between the high and low DAO subsets at the 2.0 Å threshold rather than just 23 points.

Recall the earlier discussion regarding alternative weightings of the eSim components. There was a slight performance advantage for the combination of Coulombic and hydrogen-bonding components over the full eSim calculation that also includes the surface-shape component. In terms of pose prediction, the full eSim approach had a four percentage point advantage at the 2.0 Å threshold for both best pose and top-scoring pose.

The ESR1 target had a wide diversity of aligned ligands, allowing for tens of thousands of alignments of a randomized subject ligand to the bound pose of a different ligand. Figure 9 shows the best prediction for the bound pose of a macrocyclic ligand (cyan), using the bound pose of a non-macrocycle as an alignment target (magenta). Apart from the conformational challenges endemic to macrocycles, it is also often the case that there is relatively little atomic center overlap between a macrocycle and a non-macrocycle that bind the same part of the same site. Here, while the macrocycle has 90% molecular overlap with the target ligand, it only has about 40% direct atomic overlap. The best pose from eSim was just 0.9 Å RMSD from the experimental coordinates (the top-scoring pose had RMSD of 1.3 Å).

At right in Fig. 9, similarity sticks are displayed that quantify the contributions of different parts of the alignment to the overall eSim score. The numbered positions correspond to the following observations:

1. The key donor shared by the two molecules is aligned well both in terms of position and directionality, resulting in maximal local donor similarity.
2. The beginning of divergence in surface shape similarity also coincides with a relatively dissimilar Coulombic field (the orange stick is very small).
3. Here, there is an area of high shape and reasonable Coulombic similarity, but where no donor or acceptor match exists between the subject and target molecule.
4. As with the matching proton on this same corresponding hydroxyl pair, the oxygen acceptors also align well and produce maximal similarity.
5. The macrocyclic ring closure maintains high surface shape similarity despite lacking precise atomic overlaps.
6. There are no similarity sticks where the phenyl of the target is not covered by the subject ligand, and this protrusion does not cause a shift in alignment away from allowing congruent surfaces to match.

Importantly, this type of calculation can be done in interactive time-scales, using modest multi-core workstations (see Methods). Using the ForceGen (*-pgeom*) conformer search method [25, 26], generating the conformer pool for the macrocycle shown in Fig. 9 took just 11 s of real time, and the eSim alignment took less than 0.5 s.

**Fig. 10:**
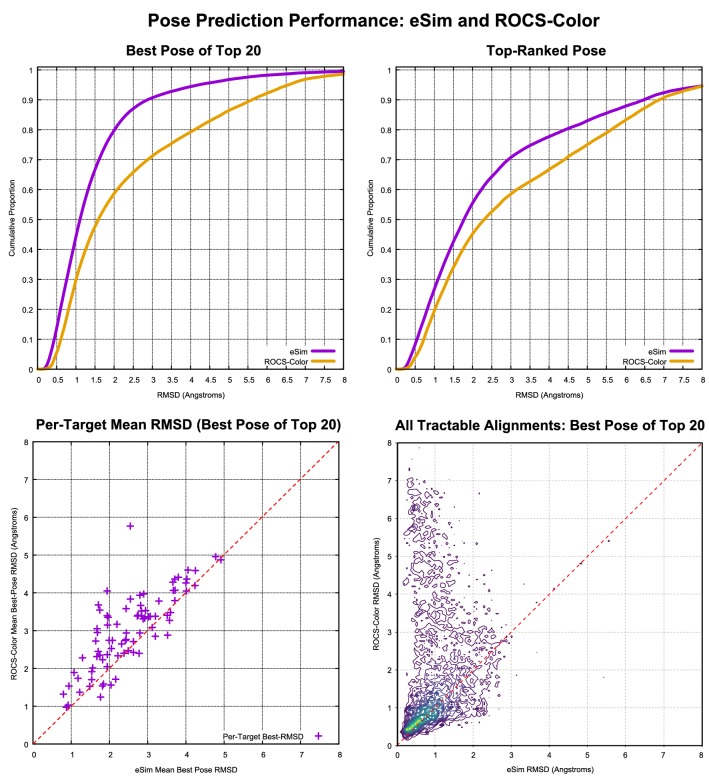
Comparison of pose prediction performance of eSim and ROCS-Color: cumulative histograms of RMSD for best pose of top 20 (top left: eSim in violet, ROCS-Color in gold); cumulative histograms of RMSD for top-ranked (top right: eSim in violet, ROCS-Color in gold); per-target average performance for best pose of top 20 (bottom left); and a contoured plot of the best-pose performance for the two method on the full data set (bottom right)

We are not aware of any comparable pose prediction benchmarks where it would have been possible to make a direct comparison to other methods. Because ROCS-color was, by a significant margin, the best of the alternative methods, it was tested on the DUD-E$^+$ benchmark in order to provide context for the eSim pose prediction performance. Testing was done exactly as with eSim, using 250 conformers per subject ligand, beginning from the same agnostic starting point in each case (see Methods for details). Figure 10 shows four plots that summarize the overall comparative performance of the two methods.

The plot at top left is analogous to that seen at the top of Fig. 8, with cumulative histograms of results for the best pose of the top 20 returned for eSim (violet curve) and ROCS-color (gold curve). At the 2.0 Å RMSD threshold, there was a roughly 20% point higher success rate for the eSim approach. The performance differential for the top-ranked pose was smaller, with a roughly 10 percentage point advantage for eSim.

For mutual alignment of ligand *sets*, differences in the best of top N RMSD performance are critical because they are multiplicative in effect. The probability of observing a close-to-correct alignment clique among N ligands falls off in proportion to the probability of the constituent pairwise comparisons containing correct relative alignments.

Note that the overall cumulative histograms reflect a greater contribution from targets with very large sets of pairwise comparisons (11 targets had over 10,000 comparisons each) than those with small sets (62 targets had 1000 or fewer comparisons). The lower left plot shows the per-target average best-pose performance for the DUD-E$^+$ 85 targets having from 20 to 60,000 ligand pose pair comparisons. In all 54 cases where the mean performance values differed by at least 0.4 Å, eSim produced the better RMSD performance value.

The lower right plot is the analogous one, but using each data point individually, rather than grouping by target. The plot was done using contoured density due to the nearly 400,000 points in the plot. Where both methods produced reasonable performance (lower left of the plot), eSim yielded quantitatively better RMSD values, with the yellow “ridge” being above the dashed line of equivalent performance. Note also that in a non-trivial fraction of cases where eSim produced results of 2.0 Å or better, the corresponding ROCS-color values were from 3.0 to 8.0 Å RMSD.

In all of the comparisons, whether assessed per-target or over all of the pose predictions made, the eSim performance advantage was highly statistically significant (p $\ll 10^{-10}$, by paired t-test).

To explore whether the performance gap between ROCS-color and eSim was mainly a property of the respective similarity functions or pose optimization procedures, a more thorough ROCS procedure was tested on a limited number of pairwise alignments (about one quarter of the full set). With the “-subrocs true” flag set, ROCS alignment sampling and optimization is vastly increased (consequently increasing computational time). Overall, the additional sampling made only a small improvement to the default ROCS procedure’s results, much smaller than the roughly 20% point gap at the 2.0 Å RMSD threshold for best of top 20 performance.

## Conclusions

We have introduced a new 3D molecular similarity method, called eSim. It was developed by analogy to the newly introduced QuanSA 3D QSAR method [17]. It compares molecular surfaces to characterize shape similarity rather than the much more widely used approach of volumetric comparison. In addition, it makes use of a physically realistic means to compare ligands’ ability to make electrostatic interactions and specific hydrogen bonds. The former is handled by direct calculation and comparison of electrostatic fields, and the latter is handled by considering the presence, precise surface position, and directional preference for each hydrogen bond donor and acceptor. The eSim calculation is asymmetric, measuring the extent to which a subject molecule is able to mimic a target.

Through extensive benchmarking using the full, unmodified DUD-E data set, we compared eSim with seven alternative methods for which performance data were available. Performance of eSim in all three screening modes was significantly better than the best-performing alternative (p < 10$^{-3}$, 10$^{-9}$, and 10$^{-12}$, respectively, from fastest to most thorough mode). In its most accurate mode, eSim had AUC values of at least 0.80 for 43% of the DUD-E targets (more than twice as many as the best-performing alternative). In its fastest mode, eSim processed roughly 275 molecules per second using a single-core/single-thread CPU calculation, roughly 5–20 times faster than the remotely competitive alternative approaches.

We have augmented the DUD-E set by comprehensively accumulating data from additional PDB structures. This allowed for experiments involving alternative query choices and a very extensive data set for assessing ligand-based pose prediction. Using multiply-aligned ligands as joint targets for similarity-based screening, eSim obtained a mean AUC of 0.82 across the 92 DUD-E$^+$ targets, which was equal to the performance obtained using the optimal single ligand as a query. Pose prediction results were encouraging for the prospects of accurate multiple-ligand alignment. In 80% of the nearly 400,000 pairwise ligand alignment cases where some reasonable degree of shared bound volume existed, a predicted pose was returned within 2.0 Å RMSD of experimental coordinates.

The nominal performance advantage of eSim compared with the best alternative method (ROCS-color) in terms of screening enrichment was roughly a 14% increase in ROC AUC using comparable parameterizations. For pose prediction, the improvement in success rate at the 2.0 Å RMSD threshold was more than 30% for best returned pose and over 20% for top-ranked pose. The success rates observed suggest that purely ligand-based methods may be effective for predictive analysis of ligand binding modes, which remains a critical problem in molecular design and is a key challenge for 3D-QSAR approaches.

Future work will involve systematic exploration of the multiple alignment problem, further parametric and speed optimizations within the eSim calculation, and a GPU-based implementation.
